# Comparison of changes in the dental transverse and sagittal planes between patients treated with self-ligating and with conventional brackets

**DOI:** 10.1590/2177-6709.25.1.047-055.oar

**Published:** 2020

**Authors:** Javier Moyano, Diana Montagut, Ramon Perera, Javier Fernández-Bozal, Andreu Puigdollers

**Affiliations:** 1 Universitat Internacional de Catalunya, School of Dentistry, Department of Orthodontics (Barcelona, Spain).; 2 Private practice (Almeria, Spain).; 3 Private practice (Tarragona, Spain).

**Keywords:** Self-ligating, Conventional, Transverse, Sagittal

## Abstract

**Introduction:**

Several advantages have been established regarding the efficiency of self-ligating brackets (SL). In spite of some controversy surrounding this question in the literature, clinical results confirm that “arch development” requires fewer extractions.

**Objective:**

The objective of this study was to compare changes in the transverse and sagittal planes in patients treated with conventional ligating brackets (CL)as well as in patients treated with SL brackets and oversized arches.

**Methods:**

A sample was selected from a pool of 300 consecutive cases treated by a single clinician: 51 patients with SL brackets and oversized wires, and 55 with CL brackets. These two groups were compared with a control group of 20 patients. All plaster models were scanned and dental landmarks were measured to identify changes from commencement (T_0_) to conclusion (T_1_) of treatment. Lateral cephalograms were analyzed for changes in the lower incisor (IMPA) and the first lower molar distal angulation (MAng). Intraoperator reliability was tested with linear regression analysis. To assure all groups were comparable at T_0_, an ANOVA test with a 95%confidence interval (CI) was performed for all values. To assess changes from T_0_ to T_1_ in all groups, a Student’s t-test with 95% CI was used. Finally, results from the three groups were compared using an ANOVA-test (95% CI) and a *post-hoc* test.

**Results:**

Increases in all the transverse variables were recorded in the two groups treated (SL and CL), except for the lower intercanine distance in the SL group. IMPA difference from T_0_ to T_1_ was higher in the CL group, and molar distal angulation (MAng) took place in the SL group.

**Conclusions:**

Self-ligating brackets with oversized arches and conventional ligating brackets showed increases in all variables in the transverse plane, except for the SL group at the mandibular intercanine distance. In comparison with the CL group, fewer different IMPA values were observed in the SL group, in which distal molar angulation occurred.

## RESUMO

**Introdução:**

Já foram enumeradas diversas vantagens quanto à eficiência dos braquetes autoligáveis (BAL). Apesar das controvérsias na literatura, resultados clínicos confirmam que o “desenvolvimento da arcada” demanda menos extrações.

**Objetivo:**

O objetivo deste estudo foi comparar as mudanças nos planos transversal e sagital em pacientes tratados com braquetes convencionais (BC) e pacientes tratados com BAL e arcos superdimensionados.

**Métodos:**

A amostra foi selecionada de um grupo de 300 pacientes tratados consecutivamente por um mesmo ortodontista: 51 pacientes com BAL e arcos superdimensionados, e 55 com BC. Esses dois grupos foram comparados a um grupo controle (GC) de 20 pacientes. Todos os modelos de gesso foram escaneados e pontos de referência dentários foram mensurados, para se identificar as mudanças entre o início (T_0_) e o fim do tratamento (T_1_). Radiografias cefalométricas laterais foram analisadas para identificar mudanças nos incisivos inferiores (IMPA) e na angulação distal dos primeiros molares inferiores (MAng). A confiabilidade intraexaminador foi testada por meio de uma análise de regressão linear. Para garantir que os grupos fossem comparáveis em T_0_, realizou-se o teste ANOVA com intervalo de confiança (IC) de 95% para todos os valores. Para avaliar as mudanças entre T_0_ e T_1_ em todos os grupos, foi utilizado o teste *t* de Student com IC de 95%. Por fim, os resultados dos três grupos foram comparados utilizando-se o teste ANOVA (IC 95%) e um teste *post-hoc*.

**Resultados:**

Foram registrados aumentos em todas as variáveis transversais nos dois grupos tratados (BC e BAL), com exceção da distância intercaninos inferiores no grupo BAL. As maiores diferenças de T_0_ para T_1_ ocorreram no IMPA do grupo BC e na angulação distal de molares (MAng) do grupo BAL.

**Conclusões:**

Ambos os grupos, BAL com arcos superdimensionados e BC, apresentaram aumento em todas as variáveis no plano transversal, com exceção da distância intercaninos inferiores no grupo BAL. Em comparação ao grupo BC, menores diferenças nos valores de IMPA foram observadas no grupo BAL, no qual aconteceu angulação distal de molares.

## INTRODUCTION

When orthodontists plan a treatment, they can only resort to expansion, protrusion, distalization, extraction and/or IPR (interproximal reduction). However, patients began to demand shorter treatments without the need for extractions. In this context, self-ligating (SL) brackets have gradually seen a relaunch over the last 15 years. Although the self-ligating system was first described in 1935, brackets and different shapes of wires made of the latest alloys continue to be developed. Indeed, these systems have grown exponentially from 8.7% in 2002 to 42% in 2008.^1^ In spite of limited research showing the advantages of SL brackets over conventional ones, a number of papers have claimed that these brackets produce faster dental movements with less or no need for extractions, facilitating easier treatments and stable results.^2^ A review of well-designed RCTs (Randomized Control Trial) have only demonstrated that this system shortens chair time and produces less protrusion of the mandibular incisor, without mentioning the many benefits of SL brackets.^3^ In addition, recent systematic reviews^4^ have not shown any clinical advantages in arch expansion, space closure or orthodontic efficiency. To identify these benefits, it is necessary to compare passive SL brackets with conventional brackets to ascertain their true effect in the sagittal and transverse planes, and then match these findings with a control group. A combination of transverse and sagittal variables was studied to determine the existence of the “lip bumper effect”^2^, that is, the liberation of the dentition from the perioral muscles, which allows the dental arches to develop due to the effect of the low tongue protrusion forces. Thus, the aim of this study was to compare the transverse and sagittal planes in patients treated with passive self-ligating brackets and oversized arches, as well as in patients treated with conventional brackets.

## MATERIAL AND METHODS

### Material

This retrospective study was approved by the Ethics Committee of the *Universitat Internacional de Catalunya*. The sample was selected from a pool of 300 consecutive cases treated by a single clinician in his dental offices in Lleida and Tarragona, Spain, from 2005 to 2010. To detect a difference of 1 or more units, taking into account a standard deviation of 2, a sample of 50 patients per treated group was needed to achieve a statistical power of 80% with a significance level of 0.05. A group of 20 patients was necessary in order to identify differences and achieve a statistical power close to 100% in a control group. The sample size calculation was performed using “Sample size and power calculator GRANMO” (Institut Municipal d’Investigación Mèdica, Barcelona, Spain). The orthodontist investigator was highly experienced and has worked with conventional and SL brackets indistinctly. In accordance with this technique, the patients treated with passive SL brackets (Damon System^®^, Ormco, Glendora, Ca, USA) wore oversized wires during the first phase of the treatment. Those patients treated with conventional CL brackets (OmniArch^®^, GAC-Dentsply, Islandia, NY, USA) wore a different sequence of wires, which were customized for each patient (Table 1). Torque and angulation prescription was very similar in the two types of brackets (Table 2).

**Table 1:** Arch sequences in groups CL and SL.

CL (Roth)	SL (Damon)
0.016-in NiTi	0.014-in CuNiTi
0.018-in NiTi	0.014 x 0.025-in CuNiTi
0.016 x 0.022-in NiTi	0.018 x 0.025-in CuNiTi
0.019 x 0.025-in NiTi	0.019 x 0.025-in SS
0.019 x 0.025-in SS	

**Table 2:** Torque prescription in the two techniques.

Tooth		+1	+2	+3	+4	+5	+6	+7	-1	-2	-3	-4	-5	-6	-7
	Roth	+12^o^	+8^o^	-2^o^	-7^o^	-7^o^	-14^o^	-14^o^	-1^o^	-1^o^	-11^o^	-17^o^	-22^o^	-30^o^	-30^o^
	Damon	+12^o^	+8^o^	0^o^	-7^o^	-7^o^	-9^o^	-10^o^	-1^o^	-1^o^	0^o^	-12^o^	-17^o^	-30^o^	-10^o^

Inclusion criteria were: available records from the beginning to the end of orthodontic treatment; patients with permanent dentition; patients who only wore brackets without auxiliary appliances for transverse or sagittal plane; dental Class I or mild Class II^5^; low to moderate crowding (0-5mm); and with no prior extractions or need for them or changes in dental anatomy either. Exclusion criteria were: patients with missing teeth or those requiring extraction; severe crowding; auxiliary appliances in transverse or sagittal plane, such as expanders or Class II appliances; and full Class II or III malocclusion.

Three groups were studied: 1) SL group, consisting of 51 patients (36 women/15 men), with a mean age of 19.9 ± 11.3 years, treated with self-ligating brackets and oversized arches; 2) CL group, consisting of 55 patients (34 women/21 men), with a mean age of 16.38 ± 9.86 years, treated with conventional brackets; 3) CT group, consisting of 20 individuals (12 women/8 men), with an approximate age of 24.05 ± 2.15 years. The control group was made up of students of Dentistry (Universitat Internacional de Catalunya, Spain) with no previous orthodontic treatment, who met all the inclusion criteria. Orthodontic records of this group were taken (T_0_) and repeated at 24 months (T_1_).

### Methods

Sagittal and transverse values were measured by obtaining plaster casts of the patients prior to (T_0_) and upon completion of treatment (T_1_).Two casts, taken without any appliances on the teeth, were scanned in 1:1 proportion using an HP 1315 (Hewlett-Packard Company, Palo Alto, Ca, USA) and calibrated with a millimeter ruler as a reference. All measurements were performed by a single operator blinded to the group. Calibrations and measurements of the images, obtained with Adobe Photoshop CS (Adobe Systems Incorporated, San Jose, CA, USA) showed different values in maxilla and mandible. Data were collected and stored in an Excel^®^ 2013 file (Microsoft, Redmond, Wa, USA). Sagittal values were measured with lateral cephalograms taken at T_0_ and T_1_. In order to avoid any radiological distortion, only angular values of the lower incisor to mandibular plane (IMPA) and the molar distal angulation to the mandibular plane (MAng) were measured. Mandibular arch depth (ArchD) was determined using the same procedure as that used for all variables in the transverse plane (Table 3).

**Table 3:** Variables in the transverse plane.

Maxillary intercanine distance (DCMax)	Distance between the vertex of the cusp of the maxillary canine and the contralateral.
Maxillary first premolar distance (DP1Max)	Distance between the vertex of the vestibular cusp of the maxillary first premolar and the contralateral.
Maxillary second premolar distance (DP2Max)	Distance between the vertex of the vestibular cusp of the maxillary second premolar and the contralateral.
Maxillary first molar distance (DM1Max)	Distance between the vertex of the mesiovestibular cusp of the maxillary first molar and the contralateral.
Maxillary second molar distance (DM2Max)	Distance between the vertex of the mesiovestibular cusp of the maxillary second molar and the contralateral.
Mandibular intercanine distance (DCMd)	Distance between the vertex of the cusp of the mandibular canine and the contralateral.
Mandibular first premolar distance (DP1Md)	Distance between the vertex of the vestibular cusp of the mandibular first premolar and the contralateral.
Mandibular second premolar distance (DP2Md)	Distance between the vertex of the vestibular cusp of the mandibular second premolar and the contralateral.
Mandibular first molar distance (DM1Md)	Distance between the vertex of the mesiovestibular cusp of the mandibular first molar and the contralateral.
Mandibular second molar distance (DM2Md)	Distance between the vertex of the mesiovestibular cusp of the mandibular second molar and the contralateral.

The statistical analysis was divided into four different time points:

1) Intraoperator analysis. Five variables were randomly measured again at two weeks, by the same operator, followed by a linear analysis of regression.
2) Validation of the sample. To assure that the three groups were similar and comparable at T_0_, an ANOVA test with a 95% CI and comparative *post-hoc* tests (LSD)were performed, and Pearson’s chi-square test was used to analyze dichotomous variables.
3) Changes in SL, CL and CT from T_0_ to T_1_. A Student’s t-test with a 95% CI was used to observe changes after treatment in each group.
4) Comparison of changes among SL, CL and CT. An ANOVA test with a 95% CI was carried out, together with comparative *post-hoc* tests (LSD), to compare changes in the three groups.

All statistical tests were performed, in conjunction with the statistical service of the Universitat Internacional de Catalunya, using Statgraphics Plus Centurion XVI (Statistical Graphics Corp, Warrenton, Vi, USA).

## RESULTS

### Intraoperator analysis

A regression line with five variables of the first measurements and results was repeated after two weeks, by the same operator. A value of 0.985 was obtained in the correlation coefficient, with a standard error of ± 0.2. 

### Validation of the sample

No differences were observed in the men to women ratio in the three groups under study. A comparison of the age variable revealed no significant differences between SL (19.91±11.34 years) and CL (16.38 ± 9.86 years) groups. However, CT showed a higher mean age (24.05 ± 2.15 years, *p* = 0.0134). The transverse and sagittal variables studied among the three groups at the beginning of treatment showed no statistically significant differences (T_0_, Table 4).

**Table 4:** Variables at beginning of treatment (T_0_).

Variable	Age’	Gender”	DCMax §	DP1Max §	DP2Max §	DM1Max §	DM2Max§	DCMd§	DP1Md§	DP2Md§	DM1Md§	DM2Md§	IMPA˜	MAng˜	ArchD§
SL	19.91 ± 11.34	36/15	33.2 ± 2.08	39.36 ± 2.97	44.29 ± 2.98	49.49 ± 2.68	54.85 ± 3.24	25.66 ± 2.60	32.42 ± 2.63	37.99 ± 2.80	43.32 ± 2.48	48.38 ± 2.78	91.88 ± 7.03	95.60 ± 5.45	22.41 ± 1.95
CL	16.38 ± 9.86	34/21	33.54 ± 2.40	40.18 ± 2.07	45.06 ± 2.59	50.32 ± 2.68	55.86 ± 2.78	25.15 ± 1.68	32.98 ± 2.31	38.62 ± 2.58	43.59 ± 2.48	49.14 ± 2.79	92.40 ± 5.31	94.19 ± 5.72	21.40 ± 3.12
CT	24.05 ± 2.15	12/8	33.13 ± 1.90	39.82 ± 1.88	45.29 ± 2.05	50.16 ± 2.68	55.79 ± 2.44	24.72 ± 1.21	32.64 ± 1.66	38.17 ± 2.12	44.30 ± 1.90	49.80 ± 1.82	94.39 ± 6.98	93.78 ± 4.11	21.39 ± 1.88
p-value	0.0134*	0.5584^†^	0.6540*	0.2347*	0.2480*	0.2521*	0.2971*	0.1915*	0.4801*	0.4676*	0.3235*	0.1287*	0.3686*	0.3606*	0.1447*

SL = Self-ligating group; CL = conventional group; CT = control group. *ANOVA 95%; † Pearson’s chi-square test. ’ Years. ” Female/Male. § Millimeters (mm). ˜ Angle (degrees).

### Changes from T_0_ to T_1_ in SL, CL and CT

Table 5 shows a significant increase in all variables in the transverse plane, except for DCMd in SL. The largest increase was observed at the first premolar level, in both the maxilla and the mandible (DP1Max T_0_- T_1_ = 3.68 ± 2.27 mm; DP1Md T_0_- T_1_ = 3.01 ± 2.17 mm).The sagittal plane showed a significant increase in the angulation of both the lower incisors (IMPA T_0_- T_1_ = 3.15 ± 6.78^o^) and molars (MAng T_0_- T_1_ = 7 ± 4.41^o^). A significant increase in CL was noted in all the transverse plane variables, the largest of them in the second premolar area (DP2Max T_0_- T_1_ = 2.06 ± 2.73 mm; DP2Md T_0_- T_1_ = 1.90 ± 2.01 mm). In the sagittal plane, only the variable of the lower incisor angulation increased (IMPA T_0_- T_1_ = 5.53 ± 7.13^o^). However, in CT, a significant reduction occurred in the transverse variables in both the mandible and the premolar region of the maxilla. No changes were observed in the sagittal plane variables.

**Table 5:** Comparison of changes from T_0_ to T_1_ among the three groups.

Variable	Group	Mean T_0_	SD	Mean T_1_	SD	T_0_-T_1_	SD	LSD^§^	P-value^#^
DCMax (mm)	SL	33.20	2.08	35.01	1.58	1.82***	1.99	a	0.0006
	CL	33.54	2.40	34.56	1.69	1.03**	2.28	b	
	CT	33.13	1.90	32.82	1.91	-0.25 NS	0.63	c	
DP1Max (mm)	SL	39.36	2.97	43.04	1.7	3.68***	2.27	a	<0.0001
	CL	40.18	2.07	42.82	1.8	2.63***	1.94	b	
	CT	39.82	1.88	39.15	2.11	-0.55***	0.43	c	
DP2Max (mm)	SL	44.29	2.98	47.56	1.81	3.27***	2.23	a	<0.0001
	CL	45.06	2.59	47.8	2.06	2.73***	1.92	a	
	CT	45.29	2.05	44.82	2.32	-0.43**	0.65	b	
DM1Max (mm)	SL	49.49	2.68	51.96	2.05	2.47***	1.74	a	<0.0001
	CL	50.32	2.68	52.11	2.47	1.79***	1.51	b	
	CT	50.16	2.68	49.93	2.76	-0.16 NS	1.17	c	
DM2Max (mm)	SL	54.85	3.24	57.25	2.66	2.39***	1.75	a	<0.0001
	CL	55.86	2.78	56.69	3.07	0.83***	1.03	b	
	CT	55.79	2.44	55.48	2.48	-0.26 NS	0.63	c	
DCMd (mm)	SL	25.66	2.60	26.21	1.62	0.55 NS	2.32	a, b	0.04
	CL	25.15	1.68	26.06	1.29	0.91***	1.73	a	
	CT	24.72	1.21	24.37	1.13	-0.36**	0.45	b	
DP1Md (mm)	SL	32.42	2.63	35.43	1.85	3.01***	2.17	a	<0.0001
	CL	32.98	2.31	34.88	1.6	1.9***	1.85	b	
	CT	32.64	1.66	32.01	1.59	-0.55***	0.6	c	
DP2Md (mm)	SL	37.99	2.80	40.48	1.81	2.49***	2.13	a	<0.0001
	CL	38.62	2.58	40.63	1.9	2.01***	2.15	a	
	CT	38.17	2.12	37.63	2.23	-0.48**	0.67	b	
DM1Md (mm)	SL	43.32	2.48	45.07	2.1	1.79***	1.61	a	<0.0001
	CL	43.59	2.48	44.72	2.19	1.08***	1.97	b	
	CT	44.30	1.90	43.39	2.14	-0.74***	0.86	c	
DM2Md (mm)	SL	48.38	2.78	51.06	2.65	2.68***	1.66	a	<0.0001
	CL	49.14	2.79	49.64	3.03	0.5*	1.33	b	
	CT	49.80	1.82	49.08	2.01	-0.67***	0.75	c	
IMPA (degrees)	SL	91.88	7.03	95.02	8.74	3.15**	6.78	a	<0.0001
	CL	92.40	5.31	99.53	5.32	7.13***	5.53	b	
	CT	94.39	6.98	94.5	6.85	0.11 NS	0.68	a	
MAng (degrees)	SL	95.60	5.45	102.6	5.29	7***	4.41	b	<0.0001
	CL	94.19	5.72	94.85	3.97	0.66 NS	5.28	a	
	CT	93.78	4.11	94.17	3.82	0.39 NS	1.82	a	
ArchD (mm)	SL	22.41	1.95	22.17	1.46	-0.25 NS	1.41	---	0.09
	CL	21.40	3.12	22.31	1.46	0.91 NS	3.66	---	
	CT	21.39	1.88	21.26	1.95	-0.13 NS	0.46	---	

Changes from T_0_ to T_1_ Student’s t-test (NS p>0.05; * p<0.05; ** p<0.01; *** p<0.001). § Comparative post-hoc tests LSD method 95%. # P-value ANOVA 95%.

### Comparison of changes between the SL, CL, and CT

Table 5 shows changes in the transverse and sagittal variables among the three groups. The maxilla showed significant differences among the three groups, except in the second premolar (DP2Max T_0_ -T_1_). In this variable, changes in SL and CL were the same. The most significant changes in the rest of the variables were observed in SL. The same differences were noted in both the mandible and the maxilla, except for the intercanine distance (DCMd T_0_-T_1_), where no differences in SL were seen when comparing CL and CT. 

In the sagittal plane, IMPA revealed differences among the three groups. The largest increase occurred in CL (IMPA T_0_-T_1_ = 7.13 ± 5.53^o^) followed by SL (IMPA T_0_-T_1_ = 3.15 ± 6.78^o^). The angulation of the mandibular molar also exhibited differences in relation to SL, CL and CT: SL showed the greatest increase in molar distal angulation (MAng T_0_-T_1_ = 4.41 ± 7.00^o^),while CL and CT presented no difference between them. The arch depth (ArchD T_0_-T_1_) had an ANOVA p-value > 0.05, suggesting there were no changes in this variable among the three groups.

## DISCUSSION

### Discussion of the study methodology

This retrospective study analyzed three groups of consecutive patients: two groups were treated by a single clinician and compared with a third group, the control group. 

Although two different kinds of brackets and techniques were used, the torque prescription was substantially similar, except for the prescription of the lower canine (CL = -11^o^; SL = 0^o^)(Table 2). Therefore, these values may infer that the information prescription in the bracket did not produce the effects, except for the change in the lower canine, which, as anticipated, lowered the values in the DCMd. Moreover, the exclusion and inclusion criteria for the use of the auxiliary appliances in the transverse and sagittal plane, together with similar crowding in both groups, suggest that the bracket ligation system (SL or CL) and arch shape were the only variables that could have influenced the changes.

All the variables studied were dental. To observe real changes in the transverse dimension, skeletal measurements should be taken. In two papers citing measurements taken with CBCT, only one compared changes between the two kinds of self-ligating brackets, but not with conventional brackets.^6^ In the second study, measurements in both the conventional and self-ligating type were also performed with CBCT; however, the sample in that study was smaller than the sample used in the present paper, and the measurements were only taken at the beginning of the treatment and at seven months post-treatment.^7^

### Discussion of results

The self-ligating brackets and oversized arches used in the present study produced a significant expansion of all variables in the transverse plane, except for the mandibular intercanine distance. In contrast, a significant increase in all variables was observed in the conventional appliances, including the mandibular intercanine distance. It is important to note that in the case of the conventional appliance group, the orthodontist took into account the initial intercanine mandibular distance of each patient, and the torque prescription in the lower canine showed higher negative values in CL. Some studies (Table 6) have investigated variables in the transverse and sagittal plane, but none of them used a sample as large as that of the present study. Other research^7-9^ in which SL brackets without oversized arches were used showed fewer increases in the transverse plane than increases achieved with oversized wires, possibly suggesting the effect of the shape of the wires used in the technique. In this regard, other authors^6,10^ have claimed that the changes are due to the arch shape, and not to the type of bracket. Hence, further studies may confirm the results obtained in the present study. Other authors consider that besides the bracket, patient’s individual characteristics may also be responsible.^11^ All variables were observed to decrease in the control group (CT), but, when smaller than 1 mm, this decrease was not clinically perceptible. These results are in accordance with those of another study in which the arch shape was observed from 6 months to 25 and 45 years. Once the whole dentition has erupted, a minimal decrease can be expected.^12^

**Table 6:** Results from the literature.

First author (year)	Bracket	n	Transversal variables increase (mm)	Sagittal variables increase (degrees/mm)
This study	SL (passive 1)	51	3+3 (1.82±1.99***); 4+4 (3.68±2.27***); 5+5(3.27±2.23***); 6+6 (2.47±1.74***); 7+7 (2.39±1.75***); 3-3 (0.55±2.32NS); 4-4 (3.01±2.17***); 5-5 (2.49±2.13***); 6-6 (1.79±1.61***) ; 7-7 (2.68±1.66***)	IMPA (3.15±6.78***); ArchD (0.25±1.41 NS); MAng (7.00±4.41***)
	CL	55	3+3 (1.03±2.28**); 4+4 (2.63±1.94***); 5+5(2.73±1.92***); 6+6 (1.79±1.51***); 7+7 (0.83±1.03***); 3-3 (0.91±1.79***); 4-4 (1.9±1.85***); 5-5 (2.01±2.15***); 6-6 (1.08±1.97***) ; 7-7 (0.5±1.3*)	IMPA (7.13±5.53***); ArchD (0.91±3.66 NS); MAng (0.66±5.28 NS)
Pandis^16^ (2007)	SL (passive 1)	27	3-3 (1.08**); 6-6 (2.04**)	IMPA (7.41***)
	CL	27	3-3 (1.58**); 6-6 (0.43**)	IMPA (6.22***)
Scott^19^ (2008)	SL (passive 1)	32	3-3 (2.55±2.27*); 6-6 (-0.09±2.40*)	IMPA (1.73±4.06*); ArchD (-2.27±2.63*)
	CL	28	3-3 (2.66±2.33*); 6-6 (0.63±2.12*)	IMPA (2.34±3.72*); ArchD (-1.33±3.39*)
Jiang^17^ (2008)	SL (passive 1)	13	6-6 (1.42 NS)	L1-Apo (2.66 mm NS)
	CL	13	6-6 (0.65 NS)	L1-APo (1.57 mm NS)
Fleming^9^ (2013)	SL (passive 2)	29	3-3 (0.85±1.52); 4-4 (0.73±2.06); 5-5 (1.43±2.23); 6-6 (1.41±1.7 NS)	IMPA (4.41±3.19)
	CL	31	3-3 (1.17±1.77); 4-4 (1.16±1.55); 5-5 (1.72±1.80); 6-6 (0.5±1.44 NS)	IMPA (4.32±4.16)
Tecco^23^ (2009)	SL (passive 1)	20	3+3 (3.3±2.6*); 4+4 (4.4±2.5*); 5+5 (4.2±1.8*); 6+6 (2.3±1.5*)	--
	CL	20	3+3 (2.6±2.4*); 4+4 (4.3±2.1*); 5+5 (4.1±2.1*); 6+6 (2.4±2.0*)	--
Pandis^18^ (2010)	SL (passive 1)	27	3-3 (1.6**); 6-6 (2.4**)	IMPA (3.1)
	CL	27	3-3 (1.8**); 6-6 (1.0**)	IMPA (5.1)
Vajaria^14^ (2011)	SL (passive 1)	27	3+3 (1.74±3.44*); 4+4 (2.87±3.03***); 5+5 (2.77±3.19***); 6+6 (2.79±1.60***); 3-3 (2.24±1.66***); 4-4 (4.21±2.19***); 5-5 (4.35±2.53***); 6-6 (2.24±166***)	IMPA (6.09±6.94***); ArchD (1.37±1.98 NS)
	CL	16	3+3 (1.72±2.72*); 4+4 (3.44±1.80***); 5+5 (2.87±2.41***); 6+6 (0.60±2.42 NS); 3-3 (1.85±2.47*); 4-4 (3.22±2.77***); 5-5 (2.60±3.36***); 6-6 (1.85±2.47*)	IMPA (5.33±5.59***); ArchD (0.90±1.72 NS)
Pandis^24^ (2011)	SL (passive 2)	25	3-3 (1.40±0.8); 6-6 (1.9±1.3)	--
	CL	25	3-3 (2.1±1.2); 6-6 (1.5±0.9)	--
Fleming^9^ (2013)	SL (passive 1)	28	3+3 (1.97±2.16); 4+4 (4.51±2.68); 5+5 (3.96±2.51); 6+6 (1.22±2.26)	--
	SL (active 1)	31	3+3 (1.78±2.21); 4+4 (3.75±2.31); 5+5 (3.78±1.91); 6+6 (1.82±1.59)	--
	CL	28	3+3 (0.88±2.18); 4+4 (3.7±3.19); 5+5 (3.59±2.8); 6+6 (1.41±2.08)	--
Almeida^7^ (2015)	SL (passive 3)	13	4-4 (1.27±1.95*); 5-5 (2.10±1.00*); 6-6 (0.92±088*)	--
	CL	12	4-4 (1.87±2.30*); 5-5 (1.75±1.33*); 6-6 (0.46±0.77*)	--
Yu^20^ (2014)	SL (passive 2-3)	15	3+3 (1.89±1.23); 6+6 (2.36±1.20); 3-3 (0.68±1.46); 6-6 (2.14±1.56)	ArchD (2.31±1.56)
	CL	14	3+3 (1.86±1.36); 6+6 (1.12±0.87); 3-3 (0.56±1.56); 6-6 (2.06±1.68)	ArchD (1.98±1.76)
Celikoglu^13^ (2015)	SL(passive 2)	22	3-3 (0.88±1.47*); 6-6 (0.51±0.92)	IMPA (5.25±4.77***)
	CL	24	3-3 (068±1.48*); 6-6 (0.61±1.15)	IMPA (5.38±3.37***)
Anand^11^ (2015)	SL (passive 1)	37	3+3 (0.7±3.0); 6+6 (0.6±2.1); 3-3 (1.1±1.9); 6-6 (0.8±2.3)	IMPA (5.6±6.3); ArchD (2.3±3.2)
	CL	37	3+3 (-0.3±1.9); 6+6 (0.3±1.9); 3-3 (1.0±1.5); 6-6 (1.6±1.7)	IMPA (4.9±5.2); ArchD (0.5±2.3)
	SL (passive 1)	17	3+3 (1.5±3.4); 6+6 (0.3±3.2); 3-3 (1.4±1.4); 6-6 (2.2±1.4)	IMPA (1.5±4.3); ArchD (-0.2±2.5)
	CL	17	3+3 (-0.8±1.5); 6+6 (0.7±1.8); 3-3 (0.2±1.7); 6-6 (0.5±1.6)	IMPA (6.0±7.4); ArchD (0.9±3.8)

Passive SL1 (Damon System, Ormco, Glendora, Ca, USA), Passive SL2 (SmartClip, 3M-Unitek, St Paul, Mn, USA), Passive SL3 (EasyClip Aditek, Cravinhos/SP, Brazil), Active1 (Innovation, Densply GAC International, Bohemia, NY, USA).

Changes from T_0_ to T_1_ (NS p>0.05 *; p<0.05; ** p<0.01; *** p<0.001). Bold characters indicate significant differences between studied.

The increase in the lower incisor angulation was statistically significant in both SL (3.15 ± 6.78) and CL (7.13 ± 5.53) groups, and higher in CL. A number of studies^8^ ^,^ ^11^ ^,^ ^13^ ^-^ ^19^ on this variable show an increase in the IMPA in SL and CL, but no differences between either group was shown in the present study. Some of these studies^8,13,17,19^ highlight the direct relation between the initial crowding and the increase in IMPA. The present study enrolled only patients with low to moderate crowding (0-5 mm). Molar angulation (MAng) increased significantly in SL (7.00 ± 4.41^o^), while no variations were observed in CL. To our knowledge, no studies have evaluated this variable. The combination of arch expansion and distal angulation of the molar in SL may explain the smaller inclination of the lower incisor, as well as the lower values in the arch depth. The present results showed that arch depth values from T_0_ to T_1_ are very similar to those of a recent study in which no differences were found between groups.^11^ Other authors^14^ ^,^ ^19^ ^,^ ^20^ have observed a significant increase in arch depth in both groups, showing no differences between them.

### Expansion and “lip- bumper effect”

There is little mention in the literature on the “lip bumper effect”.^2,10,14,21^ Described as the liberation of the dentition from the perioral muscles, this effect facilitates the development of the dental arches, by means of the effect of low tongue forces. In SL, in which the super-elastic oversized arches were used, there was less inclination of the lower incisor, distal angulation of the mandibular molar, and expansion of the dental arches. These effects are observed in classical lip-bumper therapy.^22^ In a recent RCT on CBCT scans of active and passive brackets, the author was unable to confirm apposition and bone growth in the transverse plane.^6^ Both self-ligating brackets and oversized arches in the present study produced dentoalveolar expansion with distal molar angulation, which may be attributed to the “lip-bumper effect”, although this has not been demonstrated to date.

### Clinical considerations

The results obtained in the present study show that in patients with low to moderate crowding, the use of self-ligating brackets and oversized arches increases the transversal measurements, except for the lower intercanine distance, with less protrusion of the lower incisor. However, given the direct relation found in the literature^8^ ^,^ ^13^ ^,^ ^17^ ^,^ ^19^ between amount of crowding and incisor protrusion, it is important to note that, due to the design of the present study, these results cannot be extrapolated to severe crowding conditions. The cause of the transverse and sagittal changes found is unclear, since self-ligating brackets and oversized arches were studied together. 

## CONCLUSIONS

» Self-ligating brackets, oversized arches, and conventional ligating brackets showed an increase in all variables in the transverse plane, except for SL in the mandibular intercanine distance. The most substantial expansion was located in the first premolar in SL and in the second premolar in CL. A significant reduction in the control group was observed in all mandibular variables, as well as first and second premolars of the maxilla.
» Distal molar angulation occurred only in SL.
» No changes in arch depth were found among groups.
» The lower IMPA observed in SL might be caused by the distal angulation of the molar and arch expansion due to oversized wires.
